# ﻿*Pheidoleklaman* sp. nov.: a new addition from Ivory Coast to the Afrotropical *pulchella* species group (Hymenoptera, Formicidae, Myrmicinae)

**DOI:** 10.3897/zookeys.1104.81562

**Published:** 2022-06-10

**Authors:** Kiko Gómez, Lombart M. Kouakou, Georg Fischer, Francisco Hita-Garcia, Julian Katzke, Evan P. Economo

**Affiliations:** 1 Garraf, Barcelona, Spain Unaffiliated Barcelona Spain; 2 Station d’écologie de Lamto, Université Nangui Abrogoua, BP 28 N’Douci, Lamto, Ivory Coast Université Nangui Abrogoua Lamto Cote d'Ivoire; 3 Biodiversity and Biocomplexity Unit, Okinawa Institute of Science and Technology Graduate University, 1919-1 Tancha, Onna-son, Okinawa, 904-0495, Japan Okinawa Institute of Science and Technology Graduate University Onna-son Japan

**Keywords:** Ants, distribution range, identification keys, new species, taxonomy, Taï National Park, X–ray micro–CT, 3D digitalisation

## Abstract

In this study the taxonomy of the *Pheidolepulchella* species group is updated for the Afrotropical region and the new species *P.klaman***sp. nov.** described. It is integrated into the existing taxonomic system by an updated identification key for the whole group and an update of the known distribution ranges of its members. High quality focus stacking images are provided, with X–ray micro–CT scanned digital 3D representations, of major and minor worker type specimens.

## ﻿Introduction

The ant genus *Pheidole* has recently been the focus of extensive taxonomic attention in the Malagasy region. Recent work mainly by Salata and Fisher (2020a, b, 2021) improved the taxonomy of the genus, creating eleven new species groups and adding dozens of new species. Much less attention has been paid to the massive undescribed *Pheidole* fauna in the Afrotropical region, remaining poorly known with dozens of species waiting to be described. The only modern attempt to organise the genus in the Afrotropics defined six species groups and assigned 67 valid species and subspecies to these groups based on qualitative morphology of the worker caste (Fischer et al. 2012). Fischer et al. (2012) also revised the *P.pulchella* group containing eleven species, of which seven were newly described. However, despite this work, most of the reorganisation of Afrotropical *Pheidole* taxonomy is still ongoing, since most valid species and many more undescribed ones need to be assigned to groups, and some new groups will need to be defined. More additions to *Pheidole* taxonomy are necessary to structure and catalogue their diversity in Africa to create a better base of understanding for the global radiations of *Pheidole* ants.

Taï National Park (TNP) is the last remaining major intact block of primary forest in West Africa. It was declared a UNESCO World Heritage Site in 1982 due to exceptional richness in fauna and ﬂora. Based on several criteria including species diversity, endemism, presence of rare species and/or endangered and critical habitats, the TNP is considered a priority for the conservation of mammals, birds, amphibians, and invertebrates in West Africa (World Heritage 2022). A recent ant inventory by two of the authors (KG, LMK) in the Taï National Park at Ivory Coast yielded approximately 200 ant species and morphospecies (unpublished data), including undescribed taxa for various genera, including *Bothroponera*, *Carebara*, *Monomorium*, and *Pristomyrmex*, among others.

One of these new species is hereby described as *Pheidoleklaman* sp. nov. which we place within the *P.pulchella* group sensu Fischer et al. (2012). Since more material of the group has been sampled since Fischer et al. (2012), we take the occasion to update the illustrated identification key to incorporate the new species, as well as to provide distribution maps with updated distribution ranges for all species. The new species is described based on qualitative morphology of the worker caste, which we illustrate with high quality coloured focus stacking images and 3D models based on x-ray microtomography.

## ﻿Materials and methods

Terminology follows Bolton (1994). Measurements follow Fischer et al. (2012).

Specimens were examined under a Leica MZ16A stereo microscope and measured at × 100. All images have been modified from originals and edited using GIMP (to compose the figures), IMAGEJ (to scale and add scale bars), and Polarr Photo Editor freeware (to sharpen and improve exposure and contrast). All micro-CT scans were performed at the Okinawa Institute of Science and Technology Graduate University (**OIST**), Japan, using a Zeiss Xradia 510 Versa 3D X-ray microscope operated with the Zeiss Scout-and-Scan Control System software (v. 11.1.6411.17883) and saved in DICOM format. 3D reconstructions of the resulting scan projection data were done with the Zeiss Scout-and-Scan Control System Reconstructor (v. 11.1.6411.17883) and saved in DICOM file format. Details on scanning parameters can be found in Table 5. Postprocessing of DICOM raw data was performed with Amira software (version 6.3). Virtual examinations of 3D surface models were performed by using the ‘volren’ function. The desired volume renderings were generated by adjusting colour space range to a minimum so that the exterior surface of specimens remained visible at the highest available quality. The 3D models were rotated and manipulated to allow a complete virtual examination of the scanned specimens in Amira, and in addition, exported in PLY format to be uploaded to the online 3D model platform Sketchfab (https://sketchfab.com).

Images of shaded surface display volume renderings were made with the ‘snapshot’ function at the highest achievable resolution (usually at around 1900 × 893 pixels). Surface rendering rotational videos were created in Blender (v. 2.91, Blender Foundation, https://www.blender.org/). For optimal display, surface models exported from Amira were shaded ‘smooth’, scaled to ~ 1 m, illuminated with HDRi lighting, and rotated around their own z-axis based on specimens’ lateral views.

All specimens used in this study have been databased, and the data are freely accessible on AntWeb (2022). Each specimen can be traced by a unique specimen identifier attached to its pin. The Cybertype dataset provided in this study consist of the full micro-CT original volumetric datasets (in DICOM format), 3D surface models (in PLY formats), 3D rotation video files (in MKV format), all stacked digital colour images, and all image plates including all important images of 3D models for each species. All data have been archived and are freely available from the Dryad Digital Repository. In addition to the cybertype data on Dryad, we also provide freely accessible 3D surface models of the holotype and two paratypes of the new species on Sketchfab.

### ﻿Collection references

**NHMUK**Natural History Museum, London, UK;

**CASC**California Academy of Sciences Collection, California, USA;

**FHGC** Francisco Hita–Garcia Collection, Okinawa, Japan;

**KGAC** Kiko Gómez Abal Collection. Barcelona, Spain;

**YKPC** Yeo Kolo Collection, Lamto Station, Ivory Coast;

**RBINS**Royal Belgian Institute of Natural Sciences, Brussels, Belgium.

### ﻿Measurements and indices

Measurements and indices follow Fischer et al. (2012). All linear measurements are in millimetres (mm).

**HL** Head length. Maximum distance from the mid–point of the anterior clypeal margin to the mid–point of the posterior margin of the head, measured in full–face view; in majors from midpoint of tangent between anteriormost position of clypeus to midpoint of tangent between posteriormost projection of the vertex.

**HW** Head width. Measured at the widest point of the head, in full–face view behind eye–level.

**SL** Scape length. Maximum scape length, excluding basal condyle and neck.

**EL** Eye length. Maximum diameter of compound eye measured in oblique lateral view.

**MF** Metafemur length. Measured from the junction with the trochanter to the junction with the tibia.

**MDL** Mandible length. Maximum length, measured in oblique frontolateral view, from apex to lateral base.

**PW** Pronotal width. Maximum width of pronotum measured in dorsal view.

**WL** Weber’s length. Diagonal length of mesosoma in lateral view from the anterior point of the pronotal slope and excluding the neck, to the posteroventral margin of the propodeum.

**PSL** Propodeal spine length. In dorsocaudal view, with the apex of the measured spine, its base, and the centre of the propodeal concavity between the spines in focus: measurement is taken from apex to base along the one axis of a dual–axis micrometre, which is aligned along the length of the spine, crossing the second axis at the base of the measured spine, and the latter connecting the base with the centre of the propodeal concavity.

**PTL** Petiole length. Maximum diagonal length of petiole, measured in lateral view, from most anteroventral point of the peduncle to most posterodorsal point at the junction to first helcial tergite.

**PTH** Petiolar node height. Maximum height of petiolar node measured in lateral view from the highest (median) point of the node, orthogonally, to the ventral outline of the node.

**PTW** Petiolar node width. Maximum petiolar node width, measured in dorsal view.

**PPL** Postpetiole length. Maximum length of postpetiole, measured in lateral view, from anterior beginning of the dorsal slope to the posterior juncture of postpetiole and second helcial tergite.

**PPH** Postpetiole height. Maximum height of postpetiole, measured in lateral view, from the highest (median) point of the node to the lowest point of the ventral process, often in an oblique line.

**PPW** Postpetiole width. Maximum width of postpetiole, measured in dorsal view

**CI**Cephalic index. HW / HL * 100

**EI** Eye index. EL / HW * 100

**SI** Scape index. SL / HW *100

**MDI** Mandible index. MDL / HW * 100

**PSLI** Propodeal spine index. PSL / HW * 100

**PWI** Pronotal width index. PW / HW * 100

**FI** Metafemur index. MFL / HW * 100.

**PeI** Petiole index. PTW / PW * 100

**PpI** Postpetiole index. PPW / PW * 100

**PpWI** Postpetiole width index. PPW / PTW * 100

**PpLI** Postpetiole length index. PTL / PPL * 100

## ﻿Results

### ﻿Pheidolepulchella species group

Fischer et al. (2012) provide a diagnosis for the group, which remains unchanged with the minor correction of replacing the erroneous *P.diomandei* (nom. nud.) with *P.heliosa*. A synoptic list of the *P.pulchella* group is provided below.

*Pheidolebatrachorum* Wheeler, 1922

*Pheidolechristinae* Fischer, Hita Garcia & Peters, 2011

*Pheidoledarwini* Fischer, Hita Garcia & Peters, 2011

*Pheidoledea* Santschi, 1921

*Pheidoleglabrella* Fischer, Hita Garcia & Peters, 2011

*Pheidoleheliosa* Fischer, Hita Garcia & Peters, 2011

*Pheidoleklaman* sp. nov.

*Pheidolenimba* Bernard, 1953

*Pheidolepulchella* Santschi, 1910

= *Pheidoleniapuana* Wheeler, 1922

= Pheidolepulchellavar.achantella Santschi, 1939

*Pheidolerebeccae* Fischer, Hita Garcia & Peters, 2011

*Pheidolesemidea* Fischer, Hita Garcia & Peters, 2011

*Pheidolesetosa* Fischer, Hita Garcia & Peters, 2011

#### ﻿ Pheidole klaman sp. nov.

Taxon classificationAnimaliaHymenopteraFormicidae

364634B6-7AAA-5E1E-BB3A-5EC87B5DB692

http://zoobank.org/78E38050-CC84-4A26-B882-CEB9FD9B3521

##### Material examined.

**Figure 1.**
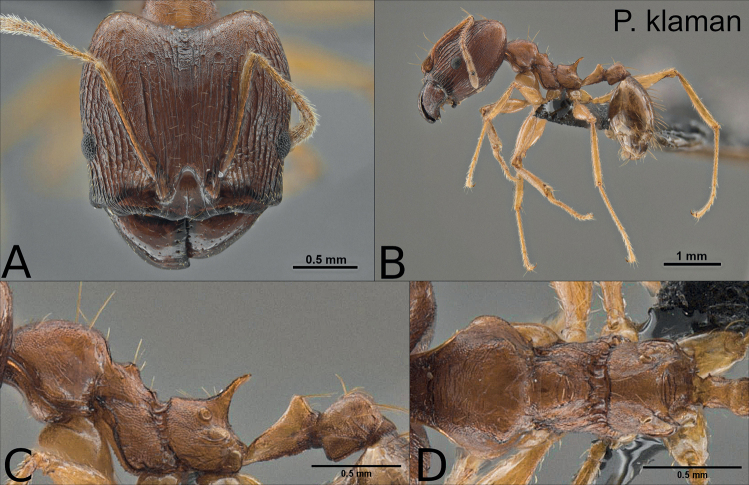
Holotype of *Pheidoleklaman*, major worker (CASENT0764691) **A** head in full-face view **B** habitus lateral view **C** mesosoma, petiole and postpetiole lateral view **D** mesosoma dorsal view.

**Figure 2.**
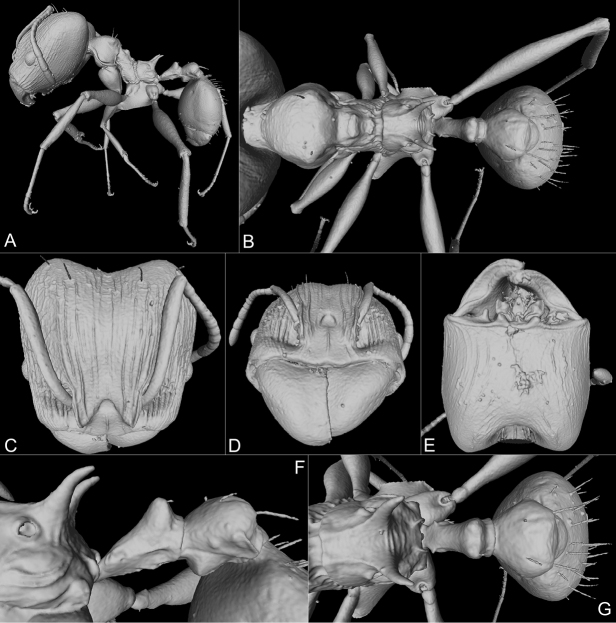
Holotype of *Pheidoleklaman*, major worker, 3D model snapshots (CASENT0764691) **A** habitus lateral view **B** habitus dorsal view **C** head dorsal view **D** head frontal view **E** head ventral view **F** propodeum, petiole and postpetiole lateral view **G** propodeum, petiole and postpetiole dorsal view.

**Major worker**: Figs 1A–D, 2A–G, 6A, B, Table 1.

**Figure 3.**
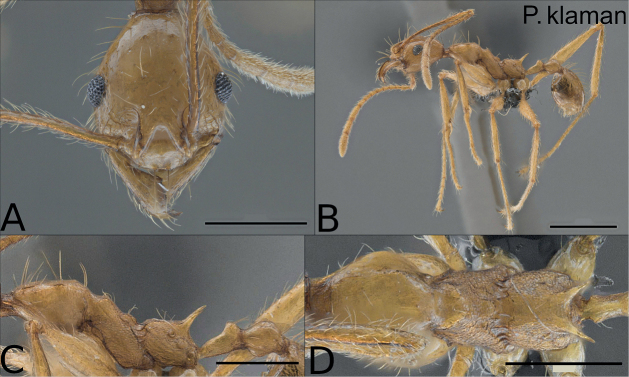
Paratype of *Pheidoleklaman*, minor worker (KGCOL00589) **A** head in full-face view **B** habitus lateral view **C** mesosoma, petiole and postpetiole lateral view **D** mesosoma dorsal view. Scale bars: 0.5 mm (**A, C, D**); 1 mm (**B**).

**Figure 4.**
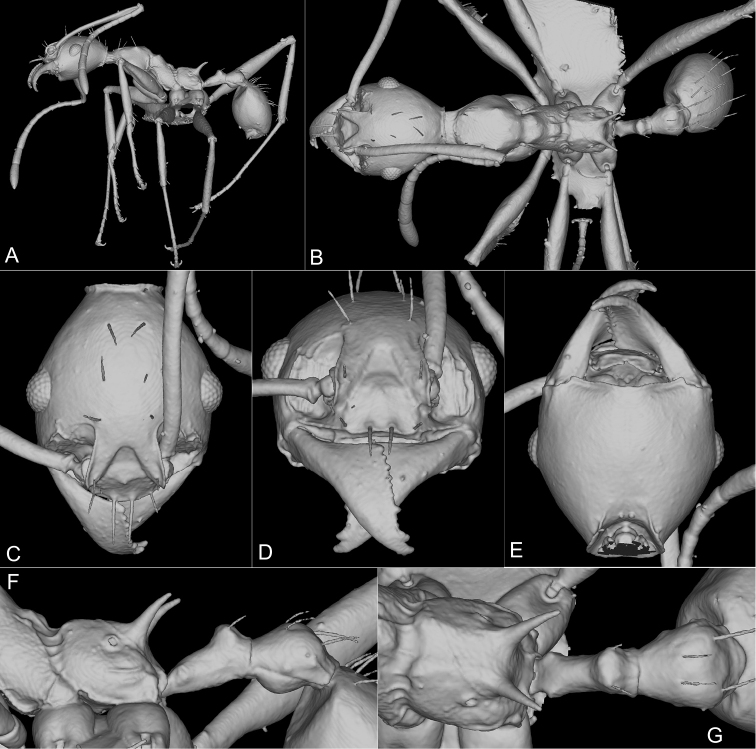
Paratype of *Pheidoleklaman*, minor worker, 3D model snapshots (KGCOL00589) **A** habitus lateral view **B** habitus dorsal view **C** head dorsal view **D** head frontal view **E** head ventral view **F** propodeum, petiole and postpetiole lateral view **G** propodeum, petiole and postpetiole dorsal view.

**Figure 5.**
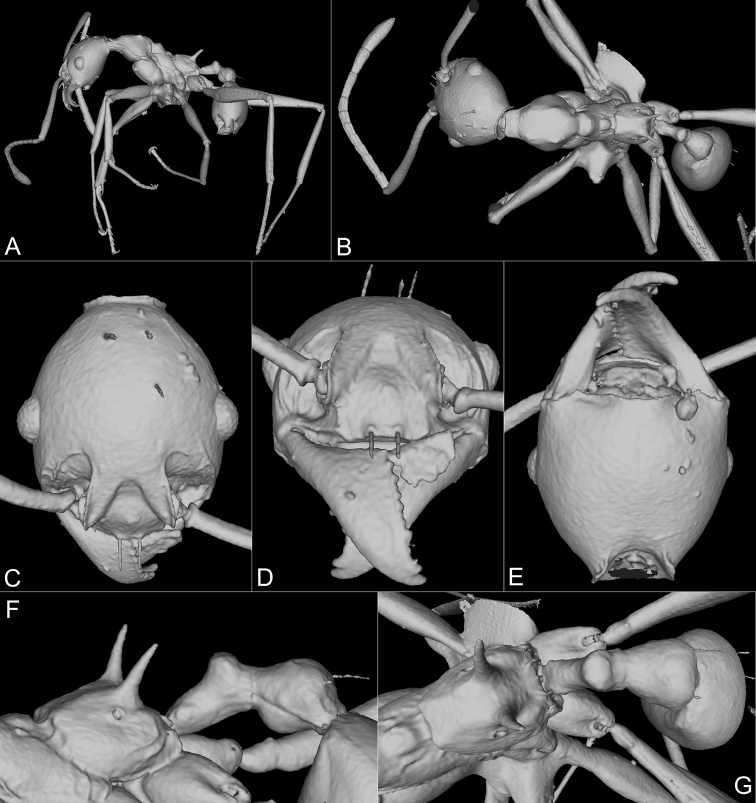
Paratype of *Pheidoleklaman*, minor worker, 3D-model snapshots (CASENT0764692). **A** habitus lateral view **B** habitus dorsal view **C** head dorsal view **D** head frontal view **E** head ventral view **F** propodeum, petiole and postpetiole lateral view **G** propodeum, petiole and postpetiole dorsal view.

**Minor worker**: Figs 3A–D, 4A–G, 5A–G, 7A, B, Table 2.

**Holotype major worker**: Ivory Coast: Montagnes District, Site 05 (Taï N. P.) 200m, 5.8438, –7.3484, 11/11/2019. Hand collected (Gómez, K. & Kouakou, L.). Primary forest, ex. rotten log [CASENT0764691] HNMUK.

**Table 1.** Measurements for the major workers of the yellow forms of the *pulchella* species group. Measurements provided as range (mean).

	Yellow species (major workers)		
	*P.heliosa* (*n* = 1)	*P.klaman* (*n* = 2)	*P.pulchella* (*n* = 12)
**HL**	2.45	1.78–1.98 (1.88)	1.94–2.23 (2.14)
**HW**	2.35	1.65–1.75 (1.7)	2.0–2.28 (2.16)
**MDL**	1.30	0.85–0.89 (0.87)	0.90–1.11 (1.02)
**EL**	0.27	0.22–0.23 (0.22)	0.23–0.26 (0.24)
**SL**	1.24	1.03–1.08 (1.06)	1.06–1.17 (1.13)
**PW**	1.07	0.74–0.78 (0.76)	0.81–1.03 (0.95)
**PSL**	0.27	0.29–0.31 (0.3)	0.24–0.38 (0.32)
**PtL**	0.61	0.45–0.47 (0.46)	0.53–0.64 (0.59)
**PtH**	0.42	0.29–0.31 (0.30)	0.33–0.39 (0.36)
**PtW**	0.31	0.21–0.22 (0.21)	0.20–0.27 (0.24)
**PPtL**	0.42	0.36–0.38 (0.37)	0.33–0.41 (0.38)
**PPtH**	0.54	0.4–0.41 (0.41)	0.37–0.5 (0.44)
**PPtW**	0.70	0.43–0.48 (0.46)	0.42–0.61 (0.52)
**WL**	2.10	1.47–1.56 (1.51)	1.40–1.74 (1.64)
**MFL**	1.98	1.44–1.51 (1.47)	1.67–1.84 (1.74)
**CI**	95	88–93 (90)	98–104 (101)
**EI**	11	12–14 (13)	11–13 (11)
**SI**	52	62–62 (62)	51–56 (53)
**MDI**	55	51–51 (51)	43–50 (47)
**PSLI**	11	17–18 (17)	11–18 (11)
**PWI**	45	45–45 (45)	39–47 (39)
**FI**	84	86–87 (87)	76–90 (81)
**Pel**	28	28–28 (28)	22–28 (25)
**Ppl**	65	58–62 (60)	47–61 (55)
**PpWI**	225	210–224 (217)	204–235 (220)
**PpLI**	145	124–126 (125)	139–166 (157)

**Paratype workers**: same series, (1 major worker) [KGCOL00585] KGAC; (1 minor worker) [CASENT0745509] FHGC; (1 minor worker) [CASENT0764692] KGAC, (1 minor worker) [KGCOL00587] AFRC; (1 minor worker) [KGCOL00588] NHMUK; (1 minor worker) [KGCOL00590] YKPC; (3 minor workers, in ethanol) [KG04152] KGAC.

***Cybertype***: we provide virtual 3D data of the major worker holotype (CASENT0764691) and two minor worker paratypes (CASENT0764692 & CASENT0745509) as cybertype dataset, which contains the following data: volumetric raw data (in DICOM format), 3D rotation videos (in mkv format), 3D surfaces (in PLY format), still images of surface volume rendering, and of stacked digital colour images illustrating head in full-face view, profile and dorsal view of the body. The datasets are deposited on Dryad (doi: 10.5061/dryad.mpg4f4r1k) and can be freely accessed as virtual representations of the types. In addition, we also provide freely accessible 3D surface models at Sketchfab (holotype: https://skfb.ly/o68Qw; paratypes: https://skfb.ly/o68Qq & https://skfb.ly/o68Qo).

##### Diagnosis.

*Pheidoleklaman* is one of the four known yellow to orange species in the *pulchella* group, and appears closest to *P.pulchella*. It is easily separable from the other yellow to orange coloured species in the group as follows:

**Table 2.** Measurements for the minor workers of yellow forms of the *pulchella* species group.

	Yellow species (minor workers)			
	*P.christinae* (*n* = 20)	*P.heliosa* (*n* = 8)	*P.klaman* (*n* = 7)	*P.pulchella* (*n* = 12)
**HL**	0.88–1.02 (0.98)	1.06–1.09 (1.08)	0.77–0.90 (0.81)	0.86–0.93 (0.89)
**HW**	0.73–0.83 (0.80)	0.78–0.82 (0.80)	0.64–0.71 (0.66)	0.73–0.80 (0.77)
**MDL**	0.58–0.68 (0.64)	0.64–0.80 (0.69)	0.55–0.62 (0.57)	0.58–0.64 (0.61)
**EL**	0.16–0.18 (0.17)	0.17–0.20 (0.18)	0.16–0.19 (0.18)	0.17–0.19 (0.18)
**SL**	1.10–1.35 (1.25)	1.30–1.43 (1.36)	1.17–1.32 (1.21)	1.13–1.24 (1.18)
**PW**	0.49–0.57 (0.54)	0.57–0.61 (0.60)	0.44–0.51 (0.46)	0.48–0.54 (0.50)
**PSL**	0.26–0.34 (0.30)	0.29–0.40 (0.32)	0.02–0.23 (0.20)	0.22–0.26 (0.24)
**PtL**	0.33–0.41 (0.38)	0.37–0.42 (0.39)	0.24–0.27 (0.26)	0.29–0.39 (0.34)
**PtH**	0.17–0.21 (0.19)	0.19–0.21 (0.20)	0.18–0.20 (0.19)	0.17–0.21 (0.19)
**PtW**	0.12–0.13 (0.13)	0.12–0.13 (0.13)	0.11–0.14 (0.12)	0.11–0.12 (0.12)
**PPtL**	0.23–0.28 (0.26)	0.27–0.28 (0.28)	0.23–0.28 (0.26)	0.21–0.26 (0.24)
**PPtH**	0.22–0.27 (0.25)	0.23–0.28 (0.25)	0.22–0.25 (0.23)	0.21–0.24 (0.22)
**PPtW**	0.22–0.27 (0.25)	0.26–0.30 (0.27)	0.24–0.27 (0.24)	0.22–0.27 (0.23)
**WL**	1.14–1.40 (1.30)	1.48–1.54 (1.51)	1.18–1.36 (1.23)	1.16–1.30 (1.25)
**MFL**	1.33–1.63 (1.49)	1.60–1.74 (1.68)	1.34–1.53 (1.40)	1.37–1.51 (1.43)
**CI**	79–84 (82)	73–75 (74)	79–83 (81)	84–90 (86)
**EI**	20–22 (21)	21–24 (22)	26–27 (27)	21–25 (23)
**SI**	144–165 (156)	163–174 (170)	182–189 (185)	146–159 (154)
**MDI**	77–84 (80)	79–99 (86)	85–87 (86)	75–82 (79)
**PSLI**	33–43 (33)	37–49 (37)	30–34 (30)	29–36 (29)
**PWI**	64–69 (64)	73–75 (73)	68–72 (68)	64–68 (64)
**FI**	175–197 (187)	205–213 (210)	208–220 (213)	179–194 (186)
**Pel**	21–25 (24)	20–23 (21)	24–29 (27)	22–24 (23)
**Ppl**	43–50 (47)	44–49 (46)	50–55 (53)	43–50 (46)
**PpWI**	183–217 (199)	208–233 (219)	186–209 (197)	183–225 (195)
**PpLI**	137–171 (149)	132–150 (139)	92–105 (100)	115–177 (146)

**Minor worker**: while *P.pulchella* has appressed scape pilosity, *P.klaman* possesses long erect setae on the scapes that are as long as or longer than the scape width. Separation from *P.heliosa* and *P.christinae* is based on size, with *P.klaman* clearly smaller (HW: 0.64–0.71) and with relatively longer scapes (SI: 182–189) than the other two species (HW: 0.73–0.83, SI: 143–174).

**Major worker**: the major of *P.christinae* is unknown. The absence of setae laterally on the head and the presence of clearly demarcated antennal scrobes separates *P.klaman* from *P.heliosa*. Separation from *P.pulchella* is also unambiguous, *P.klaman* clearly being smaller (HW: 1.65–1.75 vs 2.0–2.28 for *pulchella*), with more elongated head (CI: 88–93 vs. 98–104 in *P.pulchella*) and longer scapes (SI: 62–63 vs. 50–56). Sculpture is another separation character, with *P.klaman* being uniformly punctate from mesonotum to propodeum, versus more weakly and irregularly punctate sculpture in *P.pulchella*. In *P.klaman* the posterior third of face has faint incomplete longitudinal rugulae at most, reduced to weak, superficially reticulate punctures posteriorly, while in *P.pulchella* the posterior third of the face is longitudinally rugose and weakly to superficially punctate, changing to obliquely and weakly rugulose towards the posterior head margin.

##### Description.

With the characteristics described for *Pheidolepulchella* species group in Fischer et al. (2012) and:

**Major worker measurements: *Holotype***: HL: 1.98; HW: 1.75; MDL: 0.89; EL: 0.22; SL: 1.08; PW: 0.78; PSL: 0.31; PtL: 0.47; PtH: 0.31; PtW: 0.22; PPtL: 0.38; PPtH: 0.41; PPtW: 0.48; WL: 1.56; MFL: 1.51; CI: 88; EI: 12; SI: 62; MDI: 51; PSLI: 18; PWI: 45; FI: 86; Pel: 28; Ppl: 62; PpWI: 224; PpLI: 124

***Paratype***: HL: 1.78; HW: 1.65; MDL: 0.85; EL: 0.23; SL: 1.03; PW: 0.74; PSL: 0.29; PtL: 0.45; PtH: 0.29; PtW: 0.21; PPtL: 0.36; PPtH: 0.40; PPtW: 0.43; WL: 1.47; MFL: 1.44; CI: 93; EI: 14; SI: 62; MDI: 51; PSLI: 17; PWI: 45; FI: 87; Pel: 28; Ppl: 58; PpWI: 210; PpLI: 126

**Figure 6.**
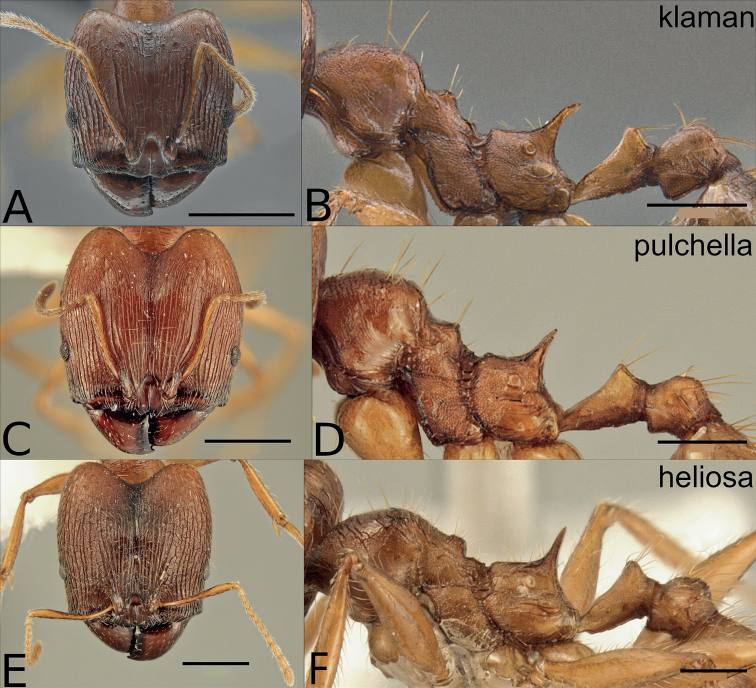
Major workers of the orange species in the *P.pulchella* group: *P.klaman* (**A, B**CASENT0764691), *P.pulchella* (C, D CASENT0218336), *P.heliosa* (**E, F**CASENT0227946). The major worker of *P.christinae* is unknown. Scale bars: 0.5 mm **B, D, F**; 1 mm **A, C, E**.

Head longer than wide (CI: 88–93), frontal carinae and antennal scrobes conspicuous. Scapes relatively long for the group (SI: 61–63). Funicular segments slightly longer than wide, the last three more than twice as long as wide, apical one longer. Pronotum shape in dorsal view rhomboid, with clearly demarcated humeral angles. Promesonotal depression and metanotal groove deep. Primary mesonotal process conspicuous, stepping into a much smaller secondary one. Propodeum dorsally with two rugulae proceeding from the metanotal groove and continuing into the well-developed, long, apically curved spines (PSLI: 17–18). Petiole with very narrow anteroventral laminar process, postpetiole with large, rounded–triangular, anteriorly oriented ventral process, in dorsal view with hexagonal to ellipsoidal shape, and two small translucent flanges extending from medial corners to gaster.

**Figure 7.**
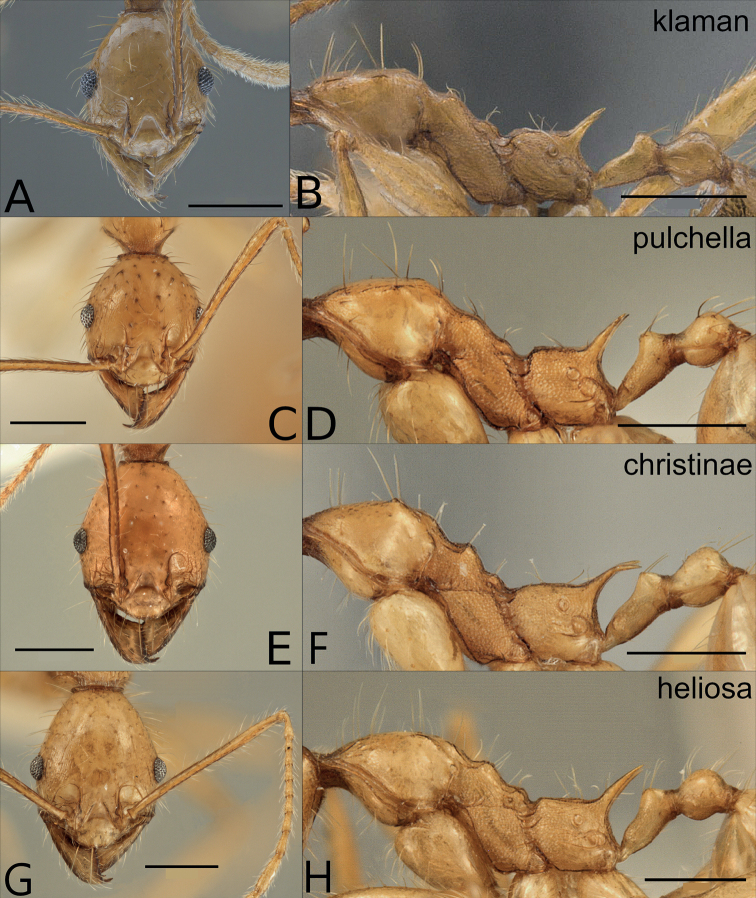
Minor workers of the orange species in the *P.pulchella* species group, head and lateral mesosoma: *P.klaman* (**A, B** KGCOL00589), *P.pulchella* (**C, D**CASENT0227963), *P.christinae* (**E, F**CASENT0227940), *P.heliosa* (**G, H**CASENT0227945). Scale bars: 0.5 mm.

Mandibles glassy smooth. Frons longitudinally rugose, with irregular pattern of moderately long to shorter rugae, spaces between rugae weakly punctate to almost smooth. Rugae grading weaker posteriorly with transverse incomplete anastomoses between them at vertex, fading from this point being gradually replaced by weaker rugulose–punctate sculpture becoming smooth at some patches. Pronotum smooth, rest of mesosoma strongly punctate, with some incomplete rugulae laterally, continuing at the base of the spines and propodeal declivity. Mesopropodeal suture with strongly demarcated longitudinal short cross–ribs. Petiole and postpetiole with the same punctuation as mesosoma, weaker to smooth dorsally. Anterior half of first gastral tergite superficially punctate, posterior half smooth.

**Table 3.** Measurements for the major workers of dark forms of the *pulchella* species group.

	Dark species (major workers)					
	*P.batrachorum* (*n* = 6)	*P.darwini* (*n* = 10)	*P.dea* (*n* = 6)	*P.glabrella* (*n* = 6)	*P.rebeccae* (*n* = 4)	*P.semidea* (*n* = 3)
**HL**	1.86–2.04 (1.91)	1.96–2.20 (2.09)	1.76–1.96 (1.83)	1.80–2.13 (1.95)	1.82–1.98 (1.92)	1.70–1.86 (1.79)
**HW**	1.80–2.0 (1.88)	1.96–2.23 (2.11)	1.78–1.94 (1.84)	1.80–2.15 (1.98)	1.86–1.98 (1.94)	1.70–1.82 (1.76)
**MDL**	0.89–1.00 (0.95)	0.94–1.06 (1.01)	0.82–0.87 (0.84)	0.78–0.97 (0.86)	0.83–0.91 (0.87)	0.79–0.83 (0.81)
**EL**	0.23–0.28 (0.25)	0.24–0.27 (0.25)	0.22–0.23 (0.23)	0.22–0.27 (0.24)	0.23–0.27 (0.26)	0.21–0.22 (0.22)
**SL**	1.01–1.13 (1.07)	1.01–1.13 (1.06)	1.01–1.06 (1.03)	0.94–1.08 (1.02)	0.94–0.98 (0.96)	0.98–1.00 (0.99)
**PW**	0.80–0.89 (0.83)	0.86–0.98 (0.93)	0.80–0.83 (0.82)	0.74–0.94 (0.86)	0.86–0.90 (0.89)	0.71–0.80 (0.77)
**PSL**	0.28–0.32 (0.31)	0.33–0.39 (0.36)	0.29–0.33 (0.31)	0.31–0.37 (0.34)	0.24–0.30 (0.28)	0.29–0.31 (0.03)
**PtL**	0.51–0.57 (0.54)	0.54–0.67 (0.59)	0.46–0.50 (0.49)	0.47–0.57 (0.53)	0.47–0.56 (0.51)	0.49–0.56 (0.52)
**PtH**	0.30–0.38 (0.33)	0.32–0.38 (0.34)	0.27–0.32 (0.30)	0.30–0.36 (0.33)	0.32–0.34 (0.33)	0.25–0.33 (0.30)
**PtW**	0.22–0.28 (0.24)	0.21–0.27 (0.23)	0.20–0.24 (0.22)	0.20–0.26 (0.23)	0.21–0.24 (0.23)	0.20–0.22 (0.21)
**PPtL**	0.33–0.39 (0.37)	0.33–0.39 (0.36)	0.29–0.32 (0.30)	0.30–0.34 (0.32)	0.31–0.36 (0.33)	0.31–0.34 (0.33)
**PPtH**	0.37–0.46 (0.41)	0.37–0.47 (0.42)	0.32–0.34 (0.33)	0.33–0.44 (0.38)	0.36–0.41 (0.39)	0.34–0.37 (0.36)
**PPtW**	0.48–0.56 (0.51)	0.47–0.59 (0.54)	0.38–0.43 (0.41)	0.41–0.54 (0.47)	0.46–0.53 (0.50)	0.44–0.47 (0.46)
**WL**	1.44–1.70 (1.54)	1.48–1.65 (1.56)	1.46–1.56 (1.51)	1.35–1.62 (1.50)	1.44–1.56 (1.50)	1.41–1.48 (1.44)
**MFL**	1.48–1.70 (1.57)	1.56–1.71 (1.62)	1.46–1.60 (1.49)	1.40–1.63 (1.51)	1.48–1.56 (1.50)	1.41–1.44 (1.43)
**CI**	97–100 (98)	99–104 (101)	99–102 (101)	99–105 (102)	99–103 (101)	98–100 (99)
**EI**	12–14 (13)	11–13 (12)	11–13 (12)	11–13 (12)	12–15 (13)	12–13 (12)
**SI**	55–58 (57)	49–53 (50)	55–58 (56)	49–53 (51)	49–51 (50)	55–58 (56)
**MDI**	47–53 (50)	42–51 (48)	42–49 (46)	42–45 (43)	44–46 (45)	43–48 (46)
**PSLI**	15–18 (15)	16–18 (16)	16–19 (16)	16–18 (16)	13–15 (13)	16–18 (16)
**PWI**	44–45 (44)	43–45 (43)	43–46 (43)	41–44 (41)	45–46 (45)	39–47 (39)
**FI**	80–85 (83)	75–80 (77)	79–83 (81)	75–78 (77)	75–84 (77)	79–83 (81)
**Pel**	27–31 (29)	24–28 (25)	25–29 (26)	24–28 (26)	24–27 (26)	25–30 (27)
**Ppl**	57–67 (62)	55–63 (58)	46–53 (50)	51–59 (55)	53–59 (56)	55–66 (60)
**PpWI**	182–236 (215)	219–257 (232)	179–200 (190)	192–220 (208)	213–230 (220)	214–224 (219)
**PpLI**	131–161 (147)	151–176 (163)	156–167 (162)	147–173 (165)	139–165 (153)	148–165 (158)

Scape pilosity appressed. In full–face view, head without standing setae laterally. Head, promesonotom, petiole, postpetiole, with several setae that are longer than spine length and with additional short appressed pubescence, long setae more abundant on gaster. Colour orange to darker orange, legs paler. Metatibia with short appressed pilosity.

**Table 4.** Measurements for the minor workers of dark forms of the *pulchella* species group.

	Dark species (minor workers)						
	*P.batrachorum* (*n* = 15)	*P.darwini* (*n* = 15)	*P.dea* (*n* = 28)	*P.glabrella* (*n* = 26)	*P.rebeccae* (*n* = 10)	*P.semidea* (*n* = 3)	*P.setosa* (*n* = 2)
**HL**	0.68–0.87 (0.82)	0.77–0.88 (0.84)	0.73–0.94 (0.85)	0.72–0.92 (0.85)	0.93–1.02 (0.97)	0.76–0.81 (0.79)	0.78–0.81 (0.79)
**HW**	0.59–0.74 (0.68)	0.67–0.76 (0.72)	0.66–0.86 (0.76)	0.67–0.86 (0.78)	0.77–0.82 (0.80)	0.72–0.78 (0.76)	0.69–0.69 (0.69)
**MDL**	0.46–0.57 (0.54)	0.52–0.61 (0.56)	0.50–0.67 (0.58)	0.51–0.63 (0.58)	0.62–0.64 (0.63)	0.51–0.56 (0.53)	0.53–0.54 (0.54)
**EL**	0.16–0.19 (0.18)	0.16–0.19 (0.17)	0.17–0.20 (0.18)	0.17–0.19 (0.18)	0.20–0.21 (0.2)	0.17–0.18 (0.17)	0.16–0.19 (0.17)
**SL**	0.9–1.13 (1.09)	0.99–1.21 (1.08)	0.94–1.16 (1.07)	0.87–1.13 (1.02)	1.27–1.32 (1.30)	0.86–0.9 0 (0.89)	0.97–1.03 (0.99)
**PW**	0.40–0.51 (0.47)	0.44–0.51 (0.48)	0.42–0.57 (0.49)	0.46–0.57 (0.51)	0.54–0.57 (0.55)	0.46–0.50 (0.48)	0.47–0.48 (0.48)
**PSL**	0.14–0.24 (0.21)	0.24–0.30 (0.27)	0.20–0.28 (0.24)	0.20–0.36 (0.28)	0.24–0.25 (0.24)	0.19–0.23 (0.22)	0.21–0.23 (0.22)
**PtL**	0.26–0.37 (0.33)	0.30–0.36 (0.33)	0.26–0.37 (0.32)	0.27–0.37 (0.34)	0.39–0.41 (0.40)	0.28–0.33 (0.31)	0.26–0.29 (0.27)
**PtH**	0.14–0.19 (0.18)	0.17–0.19 (0.18)	0.16–0.20 (0.18)	0.17–0.21 (0.19)	0.20–0.21 (0.2)	0.17–0.18 (0.17)	0.17–0.17 (0.17)
**PtW**	0.10–0.13 (0.12)	0.11–0.13 (0.12)	0.11–0.14 (0.12)	0.11–0.13 (0.12)	0.12–0.15 (0.14)	0.11–0.11 (0.11)	0.11–0.12 (0.11)
**PPtL**	0.18–0.27 (0.23)	0.19–0.24 (0.22)	0.14–0.22 (0.18)	0.18–0.23 (0.2)	0.26–0.28 (0.27)	0.19–0.22 (0.2)	0.19–0.21 (0.2)
**PPtH**	0.17–0.23 (0.21)	0.19–0.22 (0.21)	0.16–0.24 (0.20)	0.18–0.24 (0.21)	0.24–0.26 (0.25)	0.18–0.21 (0.19)	0.18–0.20 (0.19)
**PPtW**	0.19–0.24 (0.22)	0.21–0.26 (0.22)	0.17–0.24 (0.21)	0.18–0.27 (0.22)	0.26–0.30 (0.28)	0.19–0.22 (0.20)	0.21–0.22 (0.21)
**WL**	0.96–1.21 (1.12)	1.08–1.25 (1.16)	1.01–1.33 (1.16)	1.01–1.29 (1.16)	1.32–1.40 (1.36)	1.01–1.08 (1.04)	1.04–1.11 (1.07)
**MFL**	1.01–1.38 (1.28)	1.13–1.38 (1.28)	1.09–1.44 (1.27)	1.04–1.35 (1.22)	1.44–1.58 (1.51)	0.97–1.07 (1.02)	1.11–1.19 (1.14)
**CI**	80–87 (83)	84–89 (86)	86–93 (90)	88–95 (91)	81–83 (82)	94–99 (96)	85–88 (87)
**EI**	24–29 (26)	22–26 (24)	22–27 (24)	22–27 (23)	25–25 (25)	22–24 (23)	23–28 (25)
**SI**	153–173 (160)	140–159 (149)	133–146 (141)	123–140 (131)	161–165 (163)	114–121 (117)	141–149 (144)
**MDI**	76–84 (80)	75–82 (78)	73–78 (75)	71–78 (74)	78–80 (79)	68–73 (70)	77–78 (78)
**PSLI**	24–34 (24)	33–40 (33)	28–36 (28)	30–43 (30)	30–31 (30)	26–30 (26)	30–33 (30)
**PWI**	65–72 (65)	66–68 (66)	60–68 (60)	61–69 (61)	69–69 (69)	62–65 (62)	68–70 (68)
**FI**	171–197 (188)	169–190 (177)	158–175 (166)	143–169 (157)	186–192 (189)	133–139 (135)	161–172 (166)
**Pel**	24–27 (25)	22–27 (24)	22–28 (25)	22–27 (24)	23–27 (25)	22–24 (23)	23–25 (24)
**Ppl**	45–50 (48)	43–52 (46)	39–47 (42)	39–55 (44)	48–53 (50)	41–45 (43)	44–47 (45)
**PpWI**	175–200 (188)	162–217 (190)	146–192 (169)	162–209 (186)	193–208 (201)	173–200 (185)	175–200 (189)
**PpLI**	122–164 (141)	136–174 (152)	155–229 (173)	143–206 (171)	148–152 (150)	145–174 (155)	129–145 (137)

**Minor worker measurements (n = 7)**: HL: 0.77–0.90; HW: 0.64–0.71; MDL: 0.55–0.62; EL: 0.16–0.19; SL: 1.17–1.32; PW: 0.44–0.51; PSL: 0.20–0.23; PtL: 0.24–0.27; PtH: 0.18–0.20; PtW: 0.11–0.14; PPtL: 0.23–0.28; PPtH: 0.22–0.25; PPtW: 0.24–0.27; WL: 1.18–1.36; MFL: 1.34–1.53; CI: 79–83; EI: 26–27; SI: 182–189; MDI: 85–87; PSLI: 30–34; PWI: 68–72; FI: 208–220; Pel: 24–29; Ppl: 50–55; PpWI: 186–209; PpLI: 92–105

**Table 5.** Summary of micro-CT scanning parameters with resulting voxel sizes (optical magnification was 4 × and image size 1013 × 1013) for all three scanned specimens.

Subcaste	Specimen ID	Magnification	Voxel size (μm)	Exposure (s)	Power (W)	Voltage (kV)	Current (uA)
major	CASENT0764691	4 ×	5,7854	1,2	5,92	70	85
minor	CASENT0745509	4 ×	4,6952	0,6	4,01	50	80
minor	CASENT0764692	4 ×	5,3599	0,6	6,02	70	85

**Figure 8.**
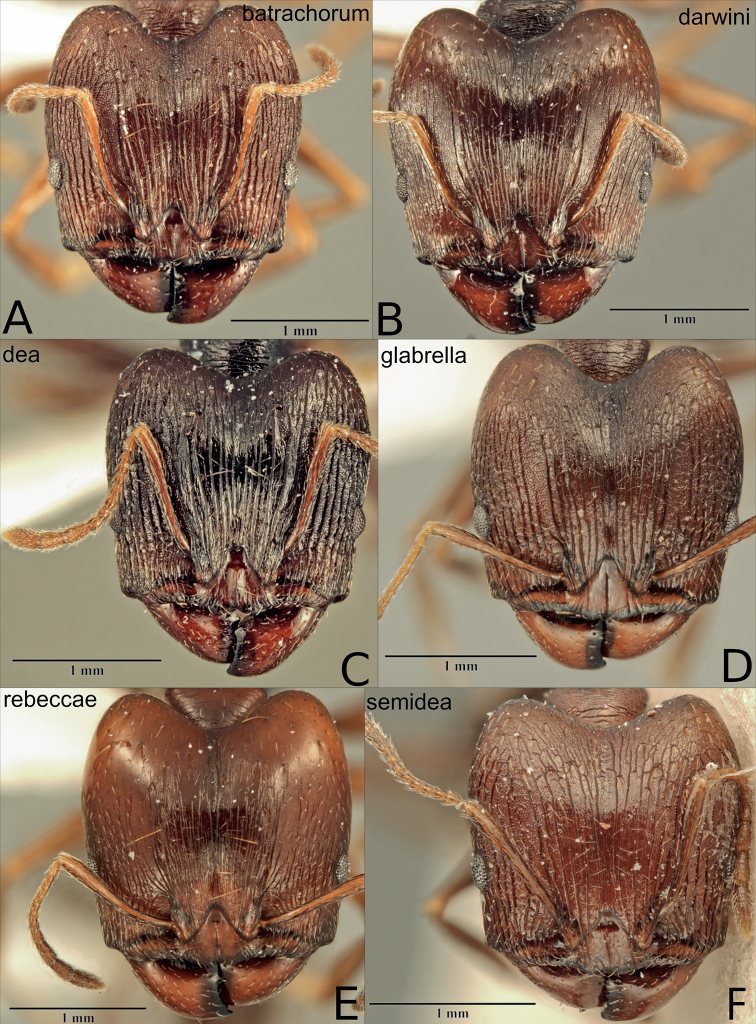
Major workers of the dark species in the *P.pulchella* group, heads in full frontal view **A***P.batrachorum* (CASENT0415367) **B***P.darwini* (CASENT0218332) **C***P.dea* (CASENT0227965) **D***P.glabrella* (CASENT0227950) **E***P.rebeccae* (CASENT0227954) **F***P.semidea* (CASENT0227960).

Head longer than wide (CI: 79–83), with sides posterior of eye level weakly convex, slightly rounded towards posterior margin. Occipital carina conspicuous, medially and laterally. Mandibles relatively long (MDI: 85–88). Scapes very long (SI: 182–189), the longest in the whole *pulchella* group. All funicular segments significantly (~ 2 ×) longer than wide, apical three segments at least 3 × as long as wide. Mesosoma as described for the group, with moderately long, apically tapering, posteriorly curved spines (PSLI: 30–34). Pronotal humeri slightly peaked in lateral view, first and second mesonotal processes notorious and pronounced, each of these structures weakly marginate, or at least with some feeble rugulae. Metanotal groove broad and deep. Legs very long (FI: 208–220), relative to body size, the longest of all the *pulchella* group.

**Figure 9.**
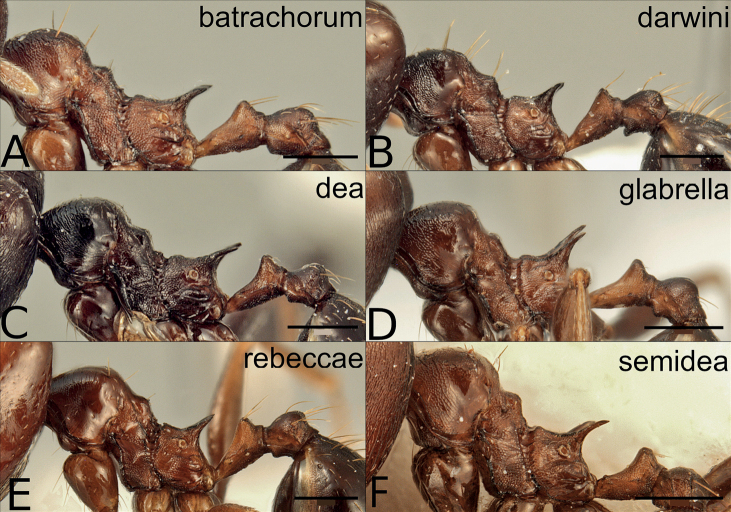
Major workers of the dark species in the *P.pulchella* group, mesosoma in lateral view **A***P.batrachorum* (CASENT0415367) **B***P.darwini* (CASENT0218332) **C***P.dea* (CASENT0227965) **D***P.glabrella* (CASENT0227950) **E***P.rebeccae* (CASENT0227954) **F***P.semidea* (CASENT0227960). Scale bars: 0.5 mm.

Head, mandibles, pronotum, coxae, legs, postpetiole, and gaster glassy smooth, except for some isolated strong reticulation between eyes and antennal fossae. Mesonotum and propodeum strongly punctate, both laterally and dorsally. Metanotal groove costate. Petiole from micropunctate dorsally to weakly punctate ventrally.

**Figure 10.**
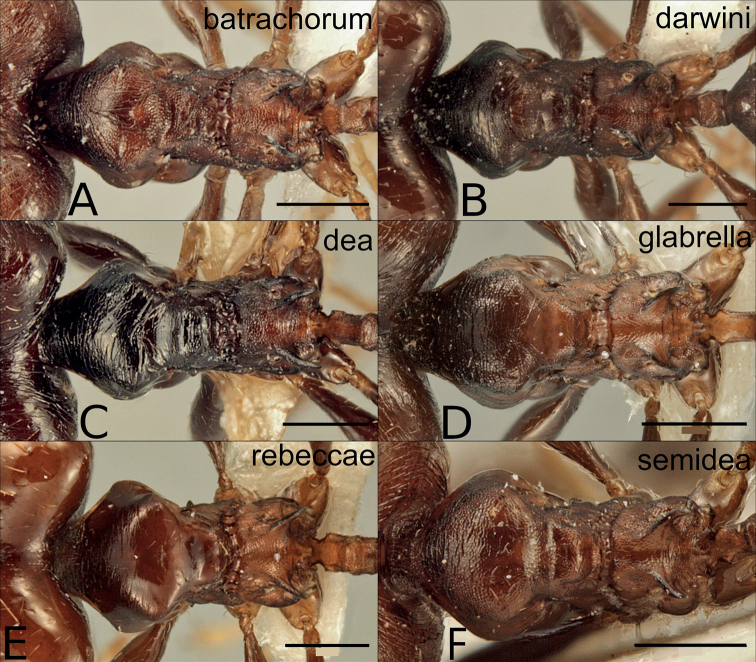
Major workers of the dark species in the *P.pulchella* group, mesosoma in dorsal view **A***P.batrachorum* (CASENT0415367) **B***P.darwini* (CASENT0218332) **C***P.dea* (CASENT0227965) **D***P.glabrella* (CASENT0227950) **E***P.rebeccae* (CASENT0227954) **F***P.semidea* (CASENT0227960). Scale bars: 0.5 mm.

Long, orange erect to semi-erect setae sparsely distributed everywhere, including pro- and mesonotum, propodeum, petiole, postpetiole, and gaster, some longer than spine length. In full-face view, head laterally with semi-erect setae above and below eyes, some longer than eye diameter. Scapes with very abundant semi-erect setae, as long as scape width. Legs also covered with abundant semi–erect to decumbent pilosity.

Colour yellow to pale orange.

##### Derivatio nominis.

The species name *klaman* is a non–Latin noun used in apposition and is the Boualé word for *pulchella* (beauty).

##### Other material examined.

Ivory Coast: Montagnes District, Site 02 (Taï N. P.) 200m, 5.8312, –7.3429 08/11/2019. Winkler sample (Gómez, K. & Kouakou, L.). Primary Forest in leaf litter (1 worker) [KGCOL00507] KGAC

Ivory Coast: Montagnes District, Site 05 (Taï N. P.) 200m, 5.8438, –7.3484 11/11/2019. Winkler sample (Gómez, K. & Kouakou, L.). Primary Forest in leaf litter (1 worker) [KGCOL00443] KGAC

### ﻿Identification key to *P.pulchella* group species

Measurements and indices are sometimes crucial for identification, so we have added detailed comparative measurements and indices for each species separated in yellow forms (Table 1 - minors, Table 2 - majors) and dark forms (Table 3 - minors,Table 4 majors).

**Minor workers**

**Table:** 

1	Colour yellow to orange	**2**
–	Colour reddish brown to black	**5**
2	Pilosity on scape and metatibia decumbent (Fig. 7C)	***P.pulchella***
–	Pilosity on scape and metatibia suberect to erect (Fig. 7A, E, G)	**3**
3	Smaller species (HW: 0.64–0.71) with relatively longer scapes (SI: 182–189)	***P.klaman* sp. nov.**
–	Larger species (HW: 0.74–0.83) with relatively shorter scapes (SI: 144–174)	**4**
4	Head, scapes and legs very long (CI: 73–75, SI: 163–174, FI: 205–213); occipital carina broadly extended and collar-like; standing hairs acute and very abundant, present also on lower meso– and metapleuron, visible in dorsal view (Fig. 7G, H)	***P.heliosa***
–	Head, scape and legs relatively shorter (CI: 79–84, SI: 144–165, FI: 175–197); occipital carina narrow, not collar-like; standing hairs often apically truncated or split, generally less abundant, absent on lower meso- and metapleuron (Fig. 7E, F)	***P.christinae***
5	Head in full frontal view laterally with several relatively long, projecting hairs posterior of eye-level (Figs 11A, B, 12D)	**6**
–	Head in full frontal view laterally completely without or at most with one or two moderately long projecting hairs near eyes or towards posterior margin (Figs 11C, D, 12A, B, C)	**8**
6	Head shape elliptical (CI: 80–89); posterior margin relatively narrow and evenly convex; occipital carina with weak median impression, scape pilosity uniformly suberect or decumbent (Figs 11A, B, 13A, B)	**7**
–	Head shape broadly rounded, posterior margin not evenly convex (CI: 86–90); occipital carina without median impression; scape pilosity decumbent with additional suberect hairs on outer edge (Figs 12D, 13H)	***P.setosa***
7	Head relatively narrow (CI: 80–87); scapes long (SI: 153–173); scape pilosity uniformly decumbent; face almost completely and distinctly punctate (Figs 11A, 13A)	***P.batrachorum***
–	Head relatively wider (CI: 84–89); scapes slightly shorter (SI: 140–159); scape pilosity uniformly subdecumbent to suberect; face smooth and shiny to very faintly punctate (Fig. 11B, Fig. 13B)	***P.darwini***
8	Sculpture variable, but head and mesosoma never completely and coarsely punctate; at least medially between eyes and on posterior dorsopronotum superficially sculptured to smooth and shiny (Fig. 11C, D, Fig. 12B, C, Fig. 14C, D, F, G)	**9**
–	Head and mesosoma almost completely and coarsely punctate (Fig. 12A, Fig. 14E)	***P.nimba***
9	Head longer than wide (CI: 85–95), posterior margin roundly or slightly convex; scapes and mandibles moderately long (SI: 123–149, MDI: 71–78) (Fig. 11C, D, Fig. 12C)	**10**
–	Head almost as wide as long (CI: 94–99), posterior margin not convex, but almost straight (Fig. 12C); scapes and mandibles shorter (SI: 114–121, MDI: 68–73) (Fig. 12B)	***P.rebeccae***
10	Long or moderately long hairs completely absent on mesosoma and waist segments; petiole and postpetiole without laterally projecting hairs in dorsal view; metatibia pilosity appressed; second mesonotal process and sculpture on propodeum reduced; metanotal groove wide in profile; spines long (PSLI: 30–43) (Fig. 14D)	***P.glabrella***
–	Moderately long hairs at least present on waist segments, sometimes also on promesonotum; on petiole and/or postpetiole some laterally projecting hairs in dorsal view; metatibia pilosity decumbent; second mesonotal process and sculpture on propodeum not reduced; metanotal groove relatively narrow in profile; spines slightly shorter (PSLI: 28–32) (Fig. 14C, G)	**11**
11	Posterior head margin roundly convex; face and dorsal promesonotum mostly superficially punctate to punctate; second mesonotal process not raised above the level of dorsopropodeum; postpetiole relatively short (PpLI: 155–229) (Fig. 11C, Fig. 14C)	***P.dea***
–	Posterior head margin weakly convex, with small median impression; face and promesonotum smooth and shiny, with very few superficial punctures; in lateral view second mesonotal process distinctly raised above the level of dorsopropodeum; postpetiole relatively longer (PpLI: 129–145) (Fig. 12G, Fig. 14G)	***P.semidea***

**Major workers**

Majors of *P.christinae*, *P.nimba*, and *P.setosa* are unknown.

**Table:** 

1	Colour yellow to orange	**2**
–	Colour reddish brown to black	**4**
2	Antennal scrobe conspicuous; sides of head without laterally projecting hairs in full–face view (Fig. 6A, E)	**3**
–	Antennal scrobe absent or inconspicuous; head in full–face view with laterally projecting hairs (Fig. 6C)	***P.heliosa***
3	Smaller species (HW: 1.65–1.75), with more elongated head (CI: 88–93) and relatively longer scapes (SI: 62). Mesonotum to propodeum strongly punctate. Face sculpture in posterior third with faint incomplete longitudinal rugulae at most, posteriorly reduced to weak, superficially reticulate punctures (Fig. 6B)	***P.klaman* sp. nov.**
–	Larger species (HW: 2.0–2.28) with broader head (CI: 98–104) and relatively shorter scapes (SI: 51–56). Mesonotum to propodeum weakly punctate. Face sculpture in posterior third longitudinally rugose and weakly to superficially punctate, posteriorly changing to obliquely and weakly rugulose (Fig. 6D)	***P.pulchella***
4	Posterolateral lobes partly smooth and shiny (Fig. 8B, E)	**5**
–	Posterolateral lobes variably sculptured, never smooth and shiny, from punctate to longitudinally rugulose (Fig. 8A, C, D, F)	**6**
5	Lateropronotum alutaceous to reticulated, sometimes with transverse rugulae (Fig. 9B). Head above the eyes rugulose, central rugulae between the frontal ridges reaching the occipital line, occipital corners alutaceous to smooth (Fig. 8B, Fig. 10B)	***P.darwini***
–	Lateropronotum glassy smooth (Fig. 9B). Head above the eyes faintly sculptured to smooth, central rugulae between the frontal ridges failing to reach the occipital line, occipital corners glassy smooth (Fig. 8E, Fig. 10E)	***P.rebeccae***
6	Scape and metatibia pilosity fine and inconspicuous, mostly fully appressed; long standing hairs absent on promesonotum (Fig. 9D)	***P.glabrella***
–	Scape and metatibia pilosity conspicuous and decumbent; standing hairs often present on promesonotum (Fig. 9A, C, F)	**7**
7	Posterolateral lobes of head longitudinally rugose, the rugulae similar to those in the rest of the head, with spaces between rugae weakly to superficially punctate (Fig. 8C)	***P.dea***
–	Posterolateral lobes of head punctate, overlain by superficial rugulae (Fig. 8A, F)	**8**
8	Central rugulae on the head running to occipital line, sometimes broken, separated apart with wide spaces between them, some transverse rugulae may appear on the upper third of the head, but without creating a reticulum (Fig. 8F)	***P.semidea***
–	Central rugulae on the head not reaching the occipital line, close together and not leaving wide spaces between them and without transverse rugulae of any kind (Fig. 8A)	***P.batrachorum***

### ﻿Notes and new distribution data for the *Pheidolepulchella* group

Distribution maps per species can be found at Fig. 15, 16.

#### Pheidoledarwini

Republic of the Congo: Env. De Makaba (par Dimonika) (Mayombe) (L. Matile) (1 worker) [EY19812] MNHN.

#### Pheidoledea

The label for type locality of this species is “Lugombe” with no additional information and Santschi (1921) cites the locality as “Congo Belgue: Lugombe”. The current official type locality is Lugombe (DRC). Congo Belgue comprised several current countries, and Lugombe is a city in Uganda, close to the capital Entebbe, so, we propose to change the type locality from Lugombe (DRC) to Lugombe (Uganda). Our new citation is the first for DRC.

**Figure 11.**
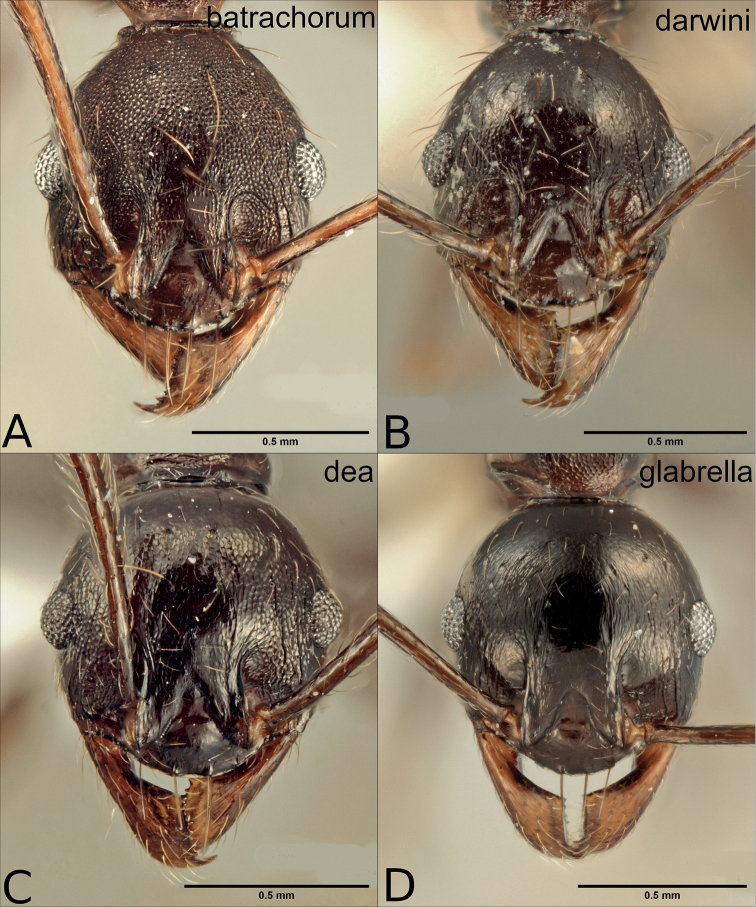
Minor workers of the dark species in the *P.pulchella* group, full frontal view **A***P.batrachorum* (CASENT0401882) **B***P.darwini* (CASENT0227962) **C***P.dea* (CASENT0227964) **D***P.glabrella* (CASENT0227951).

Democratic Republic of Congo: Secteur Nord: Mount Ngulingo, pres Nyamgaleke (Massif Ruwenzori (ex Albert NP)) 2500 m. H. Synave 17-18/08/1953 [RBINSFOR002196] RBINS

#### Pheidoleglabrella

Previously known from Western Central Africa (Cameroon, Central African Republic, Gabon and the DRC) (Fischer et al. 2012), our findings are first records for the following three countries and expand its distribution from Central to Western Africa:

**Figure 12.**
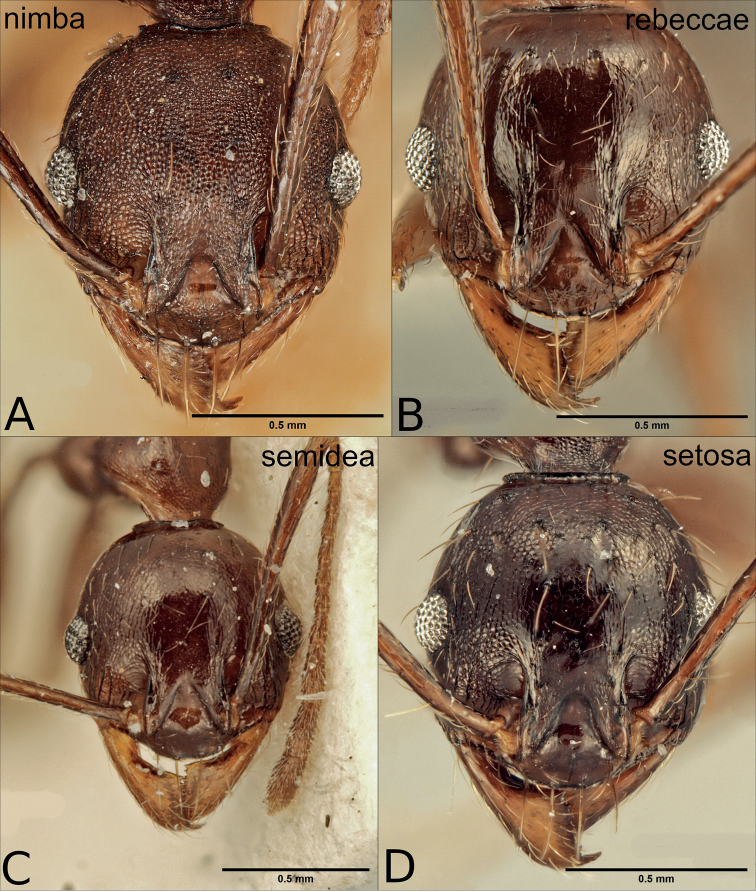
Minor workers of the dark species in the *P.pulchella* group, full frontal view **A***P.nimba* (CASENT0227967) **B***P.rebeccae* (CASENT0227955) **C***P.semidea* (CASENT0227960) **D***P.setosa* (CASENT0218298).

**Ghana**: Kumasi, FORIG, near pond, 6.7150, –1.5291, 09/01/2019. Hand collected (Gomez, K.). Fragmented degraded forest (4 workers), ex soil [KG03925C01] KGAC; same data (1 worker), [KGCOL00993] KGAC.

**Figure 13.**
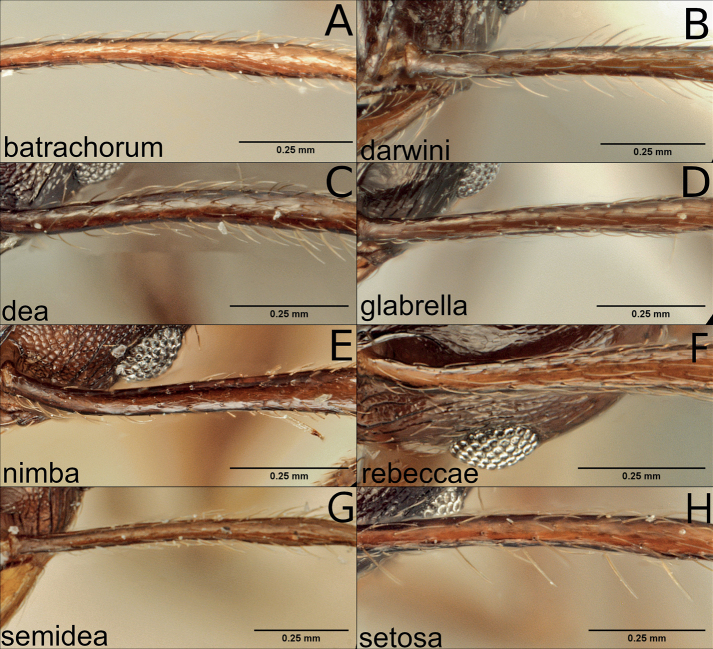
Minor workers of the dark species in the *P.pulchella* group, scapes **A***P.batrachorum* (CASENT0401882) **B***P.darwini* (CASENT0227962) **C***P.dea* (CASENT0227964) **D***P.glabrella* (CASENT0227951) **E***P.nimba* (CASENT0227967) **F***P.rebeccae* (CASENT0227955) **G***P.semidea* (CASENT0227960) **H***P.setosa* (CASENT0218298).

**Ivory Coast**: Montagnes, Mt. Tonkpi (Man), 1200 m, 7.4542, -7.6372, 23/06–01/07/2018. Malaise (Braet, Y.; Gué, A). Tropical Forest (1 worker) [KGCOL01617] RBINS.

**Figure 14.**
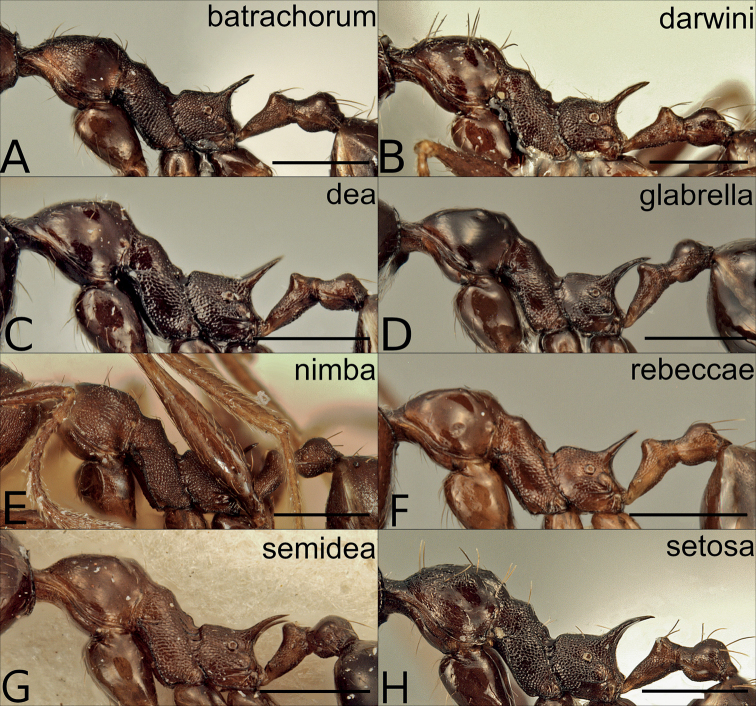
Minor workers of the dark species in the *P.pulchella* group, lateral mesosoma **A***P.batrachorum* (CASENT0401882) **B***P.darwini* (CASENT0227962) **C***P.dea* (CASENT0227964) **D***P.glabrella* (CASENT0227951) **E***P.nimba* (CASENT0227967) **F***P.rebeccae* (CASENT0227955) **G***P.semidea* (CASENT0227960) **H***P.setosa* (CASENT0218298). Scale bars: 0.5 mm.

**Republic of the Congo**: Western Cuvette, Lossi Animal Sanctuary, 0.18, 14.517, 2003. Hand collected (Rodriguez–Teijeiro, J. D.) (2 workers) [KGCOL01013] KGAC.

**Figure 15.**
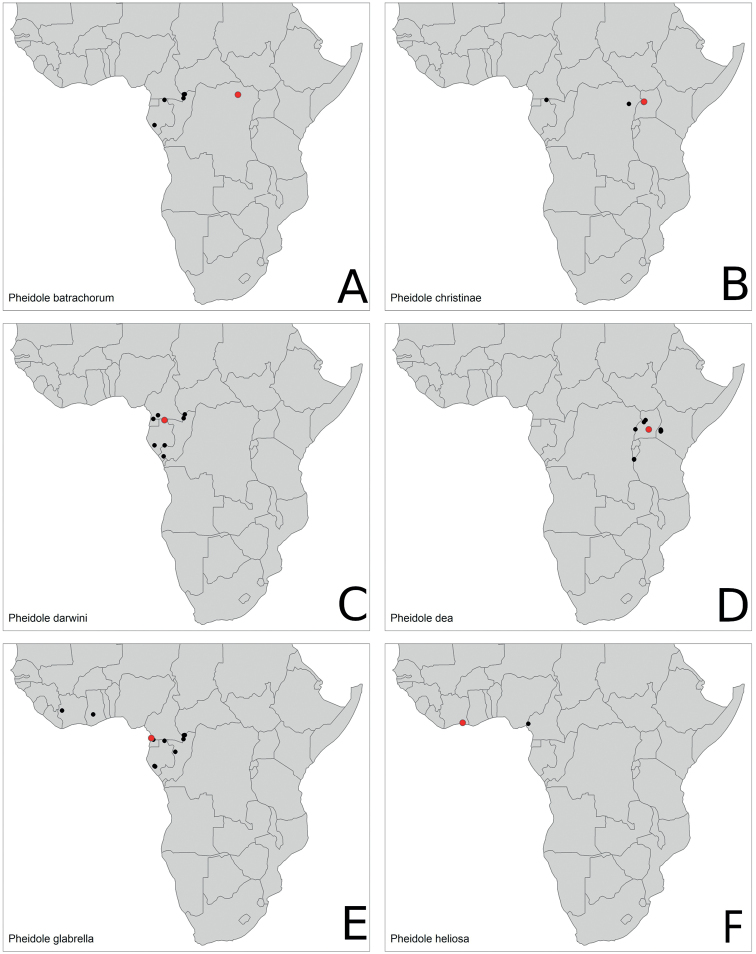
Distribution maps. **A***P.batrachorum***B***P.christinae***C***P.darwini***D***P.dea***E***P.glabrella***F***P.heliosa*. Type localities marked in red.

#### Pheidolerebeccae

Only two series are cited for this species, the type series from Ivory Coast (Abidjan) and a series from the Atewa Forest in Ghana (Fischer et al. 2012). We found it in the Taï Forest National Park in Ivory Coast, where it seems to be abundant, nesting in rotten logs:

**Figure 16.**
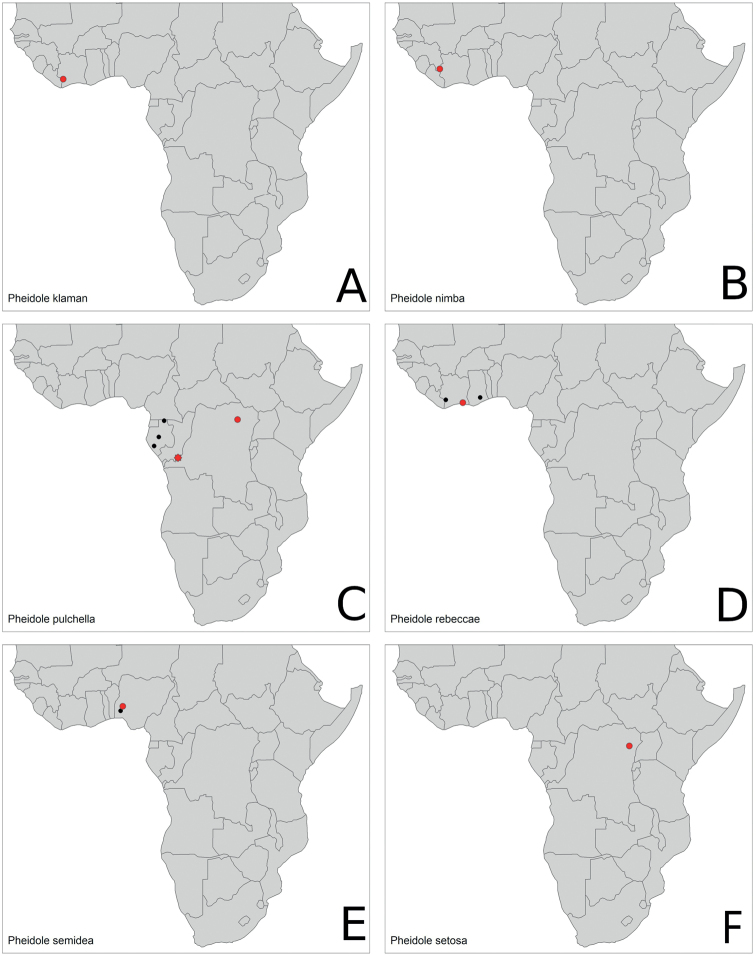
Distribution maps. **A***P.klaman***B***P.nimba***C***P.pulchella***D***P.rebeccae***E***P.semidea***F***P.setosa*. Type localities marked in red.

Ivory Coast: Montagnes District: Site 02 (Taï N. P.) 200 m, 5.8312, –7.3429 08–10/11/2019. Pitfall (Gómez, K., Kouakou, L.). Primary Forest (2 workers) [KGCOL00346] KGAC; same data, hand collected on tree trunk (1 worker), [KGCOL00725] KGAC; Site 03 (Taï N. P.) 200 m, 5.8389, –7.3452 09/11/2019. Hand collected (Gómez, K., Kouakou, L.). Primary Forest (1 worker, in ethanol), On tree trunk [KG04031C01] KGAC; same data (1 worker, pinned) [KGCOL00287] KGAC; same data, Winkler sample (2 workers, pinned) Primary forest in leaf litter [KGCOL00342] KGAC; Site 05 (Taï N. P.) 200 m, 5.8438, –7.3484 11/11/2019. Winkler sample (Gómez, K., Kouakou, L.). Primary forest in leaf litter (1 worker) [KGCOL00337] KGAC; Site 06 (Taï N. P.) 200 m, 5.8346, –7.3464 12/11/2019. Hand collected (Gómez, K., Kouakou, L.). Primary Forest (1 dealated queen, 5 majors, > 10w, in ethanol), ex. rotten log [KG04156] KGAC; same series (1 worker, 1 major, pinned), [KGCOL00511] KGAC; Site 09 (Taï N. P.) 200m, 5.8466, –7.3469 15/11/2019. Hand collected (Gómez, K., Kouakou, L.). Primary Forest (1 major, > 10 workers, in ethanol), ex. rotten log [KG04165] KGAC; same series (1 major, 1 worker, pinned) [KGCOL00330] KGAC.

## Supplementary Material

XML Treatment for Pheidole klaman

## Supplementary materials

Supplementary material 1 Video 1 Data type Video file Explanation note 3D rotation video of *Pheidoleklaman* sp. nov. holotype major worker (CASENT0764691) based on shaded volumetric surface rendering of full body. https://binary.pensoft.net/file/694542This dataset is made available under the Open Database License (http://opendatacommons.org/licenses/odbl/1.0/). The Open Database License (ODbL) is a license agreement intended to allow users to freely share, modify, and use this Dataset while maintaining this same freedom for others, provided that the original source and author(s) are credited.Kiko Gómez, Lombart M. Kouakou, Georg Fischer, Francisco Hita-Garcia, Julian Katzke, Evan P. Economo

Supplementary material 2 Video 2 Data type Video file Explanation note 3D rotation video of *Pheidoleklaman* sp. nov. paratype minor worker (CASENT0764692) based on shaded volumetric surface rendering of full body. https://binary.pensoft.net/file/694543This dataset is made available under the Open Database License (http://opendatacommons.org/licenses/odbl/1.0/). The Open Database License (ODbL) is a license agreement intended to allow users to freely share, modify, and use this Dataset while maintaining this same freedom for others, provided that the original source and author(s) are credited.Kiko Gómez, Lombart M. Kouakou, Georg Fischer, Francisco Hita-Garcia, Julian Katzke, Evan P. Economo

Supplementary material 3 Video 3 Data type Video file Explanation note 3D rotation video of *Pheidoleklaman* sp. nov. paratype minor worker (CASENT0745509) based on shaded volumetric surface rendering of full body. https://binary.pensoft.net/file/694544This dataset is made available under the Open Database License (http://opendatacommons.org/licenses/odbl/1.0/). The Open Database License (ODbL) is a license agreement intended to allow users to freely share, modify, and use this Dataset while maintaining this same freedom for others, provided that the original source and author(s) are credited.Kiko Gómez, Lombart M. Kouakou, Georg Fischer, Francisco Hita-Garcia, Julian Katzke, Evan P. Economo
